# Identification of SUV39H2 as a potential oncogene in lung adenocarcinoma

**DOI:** 10.1186/s13148-018-0562-4

**Published:** 2018-10-22

**Authors:** Yu Zheng, Baihui Li, Jian Wang, Yanjuan Xiong, Kaiyuan Wang, Ying Qi, Houfang Sun, Lei Wu, Lili Yang

**Affiliations:** 1 Department of Immunology, Tianjin Medical University Cancer Institute and Hospital, Tianjin, 300060 China; 2 National Clinical Research Center for Cancer, Tianjin, China; 3 Key Laboratory of Cancer Prevention and Therapy, Tianjin, China; 4 Tianjin’s Clinical Research Center for Cancer, Tianjin, China; 5 Key Laboratory of Cancer Immunology and Biotherapy, Tianjin, China

## Abstract

**Background:**

SUV39H2 (suppressor of variegation 3-9 homolog 2), which introduces H3K9me3 to induce transcriptional repression, has been reported to play critical roles in heterochromatin maintenance, DNA repair, and recently, carcinogenesis. Dysregulation of SUV39H2 expression has been observed in several types of cancers. However, neither the genomic landscape nor the clinical significance of SUV39H2 in lung adenocarcinoma has been probed comprehensively.

**Methods:**

In this research, we conducted bioinformatics analysis to primarily sort out potential genes with dysregulated expressions. After we identified SUV39H2, RNA-seq was performed for a high-throughput evaluation of altered gene expression and dysregulated pathways, followed by a series of validations via RT-qPCR and bioinformatics analyses. Finally, to assess the potential oncogenic role of SUV39H2, we employed the invasion assay and clone formation assay in vitro and tumorigenesis assays in mouse models in vivo.

**Results:**

Through bioinformatics analyses, we found that SUV39H2 underwent a severe upregulation in the tumor tissue, which was also confirmed in the surgically removed tissues. Overexpression of SUV39H2 was mainly associated with its amplification and with shorter patient overall survival. Then, the RNA-seq demonstrated that TPM4, STOM, and OPTN might be affected by the loss of function of SUV39H2. Finally, in vitro and in vivo experiments with SUV39H2 knockdown all suggested a potential role of SUV39H2 in both carcinogenesis and metastasis.

**Conclusions:**

SUV39H2 expression was elevated in lung adenocarcinoma. TPM4, OPTN, and STOM were potentially regulated by SUV39H2. SUV39H2 might be a potential oncogene in lung adenocarcinoma, mediating tumorigenesis and metastasis.

**Electronic supplementary material:**

The online version of this article (10.1186/s13148-018-0562-4) contains supplementary material, which is available to authorized users.

## Background

Chromatin modifications account for a major part of epigenetic regulation in mammalian cells, which are mainly composed of various posttranslational modifications (PTMs) [1], among which histone acetylation induces chromatin relaxation and therefore transcriptional activation. However, histone methylation can induce both transcriptional activation and repression. Generally, methylation of histone H3 lysine 9 (H3K9) and H3K27 is correlated with repression, and that of H3K4, with activation. Hence, under the complicated regulation processes through histone lysine methyltransferases (KMTs), lysine methylation has emerged as one of the most important PTMs in the processes from development to disease [2, 3].

One of the major KMT families that introduce transcriptional repression is the SUV39 sub-family, including SUV39H2 (KMT1B), G9a (EHMT2), G9a-like protein1, GLP (EHMT1), SETDB1 (KMT1E), and SETDB2 (KMT1F) [4], among which SUV39H1 and SUV39H2 can preferentially read H3K9me1 via their chromodomain and catalyze H3K9me3 [5–7]. Moreover, SUV39H1 and SUV39H2 exert mutually compensated expression throughout the embryonic development, implying a functional redundancy between the two enzymes [8]. However, complete knockout of both enzymes resulted in prenatal lethality, accompanied by a universal reduction of H3K9me3 levels, indicating that H3K9 methylation plays dominant roles in development [9]. Additionally, knockout of Suv39h1 and Suv39h2 could lead to abnormally long telomeres resulting from the absence of their enzymatic activities of targeting the heterochromatin [10]. Furthermore, in the dysregulation in cancer, SUV39H1 is reported to play a tumor-suppressor role due to its cell proliferation-suppressing activity [11–14]. On one hand, SUV39H2 was reported to be associated with the maintenance of heterochromatin via the introduction of H3K9me3 [15]. SUV39H2 was known to be related to DNA repair by methylating the histone H2AX [16], indicating a tumor-promoting activity. Additionally, it was found to be upregulated in bladder cancer [16], hepatocellular carcinoma [17], and acute lymphoblastic leukemia [18]. Besides, dysregulation of SUV39H2 is also seen in several other diseases. A mutation of SUV39H2 was reported to be responsible for hereditary nasal parakeratosis [19]; this provided us insights into SUV39H2-mediated epigenetic regulation in keratinocytes. Additionally, dysregulation of SUV39H2 mediated by H3K9me3 is associated with autoimmune diabetes [20] and steatohepatitis [21] in mice. The activity of SUV39H2 is modulated in various manners. It was reported that automethylation [22] and alternative splicing [23] were responsible for its specificity and function.

As stated previously, to some degree, SUV39H1 and SUV39H2 possess the opposing functions in cancer, even though they have an overlapping enzymatic activity, which might be due to the specificity of their substrate preference [24]. Identification of the target genes regulated by SUV39H2 might further elucidate the molecular mechanisms underlying its overexpression in tumor tissues. Thus, we evaluated cancer genomic data in lung adenocarcinoma and functional small-interfering RNA (siRNA), to explore the clues of SUV39H2 overexpression and its pathological activity in tumor cell lines.

## Methods

### Antibodies and reagents

The source of the anti-SUV39H2 and anti-GAPDH antibodies was Abcam, Hong Kong, China. The siRNAs were purchased from Sigma-Aldrich.

### Cell culture and transfection

All the cell lines were purchased from ATCC and maintained in the indicated media as instructed. Before culture, 10% fetal bovine serum was added to all media. Cells were cultured in a humidified incubator equilibrated with 5% CO_2_ at 37 °C. Transfection of siRNA was carried out utilizing the Lipofectamine® RNAiMAX Reagent (Invitrogen, Carlsbad, CA, USA), according to the manufacturer’s instructions.

### The Cancer Genome Atlas (TCGA) data for lung cancer

The mutation, mRNA expression, DNA copy number, and clinical data used in the research were obtained from the cBio Cancer Genomics Portal [25, 26]. The copy numbers for SUV39H1 and SUV39H2 were generated from the copy number analysis algorithm Genomic Identification of Significant Targets in Cancer (GISTIC) and classified as copy number per gene; “− 2” stands for a deep loss, “− 1” stands for a heterozygous deletion, “0” stands for neutral or diploid, “1” is a low-level gain, and “2” indicates a high-level amplification. The expression of mRNA was represented by the average reads from the RNA-seq of tumor tissues, which were obtained from TCGA [25, 26]. The value of the *Z*-scores suggests the relative deviations from the diploid samples. Somatic mutation data were obtained from exosome sequencing [25, 26].

### The Lung Adenocarcinoma (TCGA, Provisional) dataset

The Lung Adenocarcinoma (TCGA, Provisional) dataset contains approximately 520 primary lung adenocarcinoma cases with fully detailed clinical follow-up. All data were available without restrictions and limitations. For the expression and mutation analysis, the Entrez-IDs of the indicated genes were selected as the “perfect” evidence of annotation.

### The non-small cell lung carcinoma (NSCLC) in female nonsmoker (“GSE19804”) dataset

The “GSE19804” dataset (Additional file 1: Table S1) includes 120 paired tissue samples from 60 NSCLC female non-smoker patients. A detailed description of the dataset can be obtained from the original research [27]. The specimens were analyzed utilizing Affymetrix U133plus2.0 expression arrays. The analysis of the expression of the indicated genes was conducted via Illumina probes as the annotation augments.

### RT-PCR and quantitative RT-PCR (qPCR)

Total cellular RNA was obtained utilizing the Trizol reagent, following the manufacturer’s instructions (Invitrogen). cDNA was synthesized using the MMLV Reverse Transcriptase (Promega). Relative quantitation was determined utilizing the ABI PRISM 7500 sequence detection system (Applied Biosystems, Foster City, CA, USA) through the measurement of real-time SYBR green fluorescence, and the results were analyzed by means of the comparative Ct method (2^−ΔΔCt^) with GAPDH as an internal control. Each sample with the indicated primers was submitted to the PCR analyses in three replicates, and the experiment was performed in triplicate. The primers used are listed in Additional file 2: Table S2.

### ChIP-qPCR

ChIP was performed in MCF-7 cells as described previously [28, 29]. Briefly, cells were cross-linked with 1% formaldehyde, sonicated, pre-cleared, and incubated with 5–10 μg proper antibody, followed by addition of protein A/G Sepharose CL-4B beads. The beads were then washed in buffer of high and low salt concentration, and then, DNA was eluted for qPCR assays.

### Cell invasion assay

Transwell chamber filters (Becton Dickinson) were coated with Matrigel. After being transfected with siRNAs (Additional file 3: Table S3), A549 cells were suspended in serum-free 1640 medium, and then, 5 × 10^4^ cells were seeded into the upper chamber in a volume of 500 μl. The chamber was then cultured in a well containing 500 μl of 1640 media with 10% fetal bovine serum at 37 °C for 18 h. Cells on the upper side of the membrane were removed by cotton swabs and those on the other side were stained and counted. Four high-powered fields were counted for each membrane. For each complete experiment, three independent samples in the indicated groups were subjected to analysis, and every experiment was performed in triplicate.

### Tumor tissue samples

Tumor tissues were obtained from eight first-time admitted lung adenocarcinoma patients in the Tianjin Medical University Cancer Institute and Hospital (Tianjin, China), after receiving written informed consent. The utilized procedure was approved by the Ethics Committee of the Tianjin Medical University Cancer Institute and Hospital. None of the patients ever received surgery, radiotherapy, chemotherapy, or other medical intervention before the sample collection.

### Luciferase assay

The sequence of *OPTN* promoter was obtained from UCSC and cloned into pGL3-Basic for transfection. 3xFLAG-SUV39H2 and vector were transfected respectively into A549 cells, and pGL3-OPTN and Renilla were co-transfected in both groups of cells simultaneously. Both groups were harvested and examined for luciferase activity according to the manufacturer’s instruction from Promega. Each group of cells contained six independent cell cultures and each experiment was conducted in a triplicate manner.

### Xenograft assay

A549 cells stably expressing luciferase were infected with the indicated lentivirus and seeded either subcutaneously in nude athymic BALB/c mice or intravenously in immunocompromised severe combined immunodeficiency (SCID) mice, and the bioluminescence of the neoplasia was measured via the IVIS imaging system (Xenogen). Six mice were randomly assigned into each group.

### Statistical analysis

Statistical analysis was conducted via R software (http://www.r-project.org) and SPSS Statistics 22. For the data process of the GSE19084 dataset, we employed the function “affycoretools” in the R software for organizing the expression matrix from the raw data and function “limma” to determine the dysregulated genes between the paired specimens in the empirical Bayes (eBayes) manner. For the TCGA dataset, the expressions and mutations of indicated genes were paired with the clinical data in the R software. The expression sets of genes were obtained from the RNA sequencing dataset (data_RNA_Seq_v2_expression_median), and the copy number variations were from the exosome sequencing data (data_CNA). When we compared the mRNA expression of SUV39H2 and overall patient survival, the samples were divided into lower and higher expression groups based on the cutoff value obtained from the ROC curve in SPSS 22. The univariate and multivariate analyses were also performed using SPSS22 with SUV39H2 expression grouping and other indicators in the TCGA dataset.

## Results

### Overexpression of SUV39H2 in lung adenocarcinoma

**Fig. 1:**
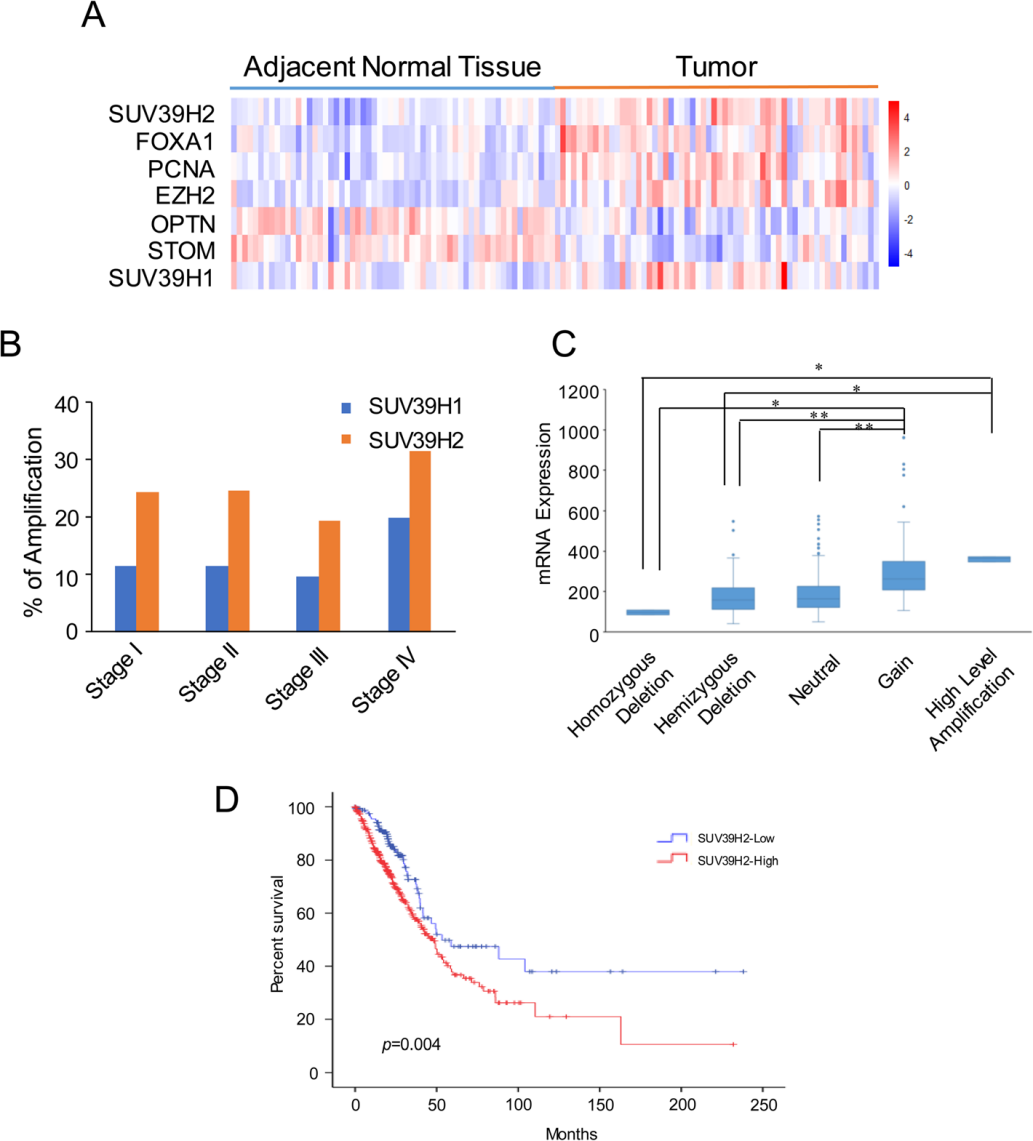
SUV39H2 was upregulated in lung adenocarcinoma. **a** The raw data of “GSE19084” chip assay was downloaded from GEO datasets and analyzed via R software. The samples were grouped into adjacent normal tissues and tumor tissues in a pair-wise manner, and the expressions of the indicated genes were analyzed in the eBayes manner. The heatmap profiled the mRNA expression of the indicated genes, and the genes with higher expression (*p* < 0.001) in the specimen are highlighted in red, and those with lower expression are highlighted in blue. **b** The CNA data of SUV39H1 and SUV39H2 were sorted from the Lung Adenocarcinoma (TCGA, Provisional) dataset of TCGA. The amplification percentages of the indicated genes in each tumor stage were analyzed according to the clinical data. **c** Different CNA statuses of SUV39H2 were plotted against the corresponding mRNA expressions of the gene, demonstrating a gradual rise as the CNA status leveled up. Correlation of each indicated pair was assessed via nonparametric test. **p < 0.01, *p < 0.05. **d** Kaplan-Meier plots of overall survival associated with the mRNA expression level of SUV39H2 in TCGA lung adenocarcinomas

To determine the expression status of SUV39H2 in lung adenocarcinoma, we analyzed the SUV39H2 expression in lung adenocarcinoma (GSE19084). The specimens were divided into the “adjacent normal tissue” and “tumor” groups, followed by statistical analysis to identify the significantly dysregulated genes in the eBayes manner (Additional file 1: Table S1). Among all the identified genes, SUV39H2 and several other classical oncogenes were prominent: FOXA1 [30, 31], PCNA [32, 33], and EZH2 [34]; additionally, we also found OPTN, which is reported to be involved in the autophagy-related apoptosis of lung cancer cells [35], and STOM, which is a membrane protein whose downregulation indicates poor prognosis and metastasis in non-small cell lung cancer [36] and HER2-positive breast cancer [37] patients. Furthermore, the expression of SUV39H1 did not differ in the two groups (Fig. 1a and Additional file 1: Table S1). DNA copy number alterations (CNAs) can result in the activation of oncogenes and silencing of tumor suppressors in human cancers [38], and CNA-derived gene dysregulation is becoming an emerging issue in lung cancer examination and treatment [39, 40]. Therefore, we hypothesized that the overexpression of SUV39H2 originated from the copy number increase of the gene. Then, we identified that the amplification percentages of SUV39H2 in different tumor stages were consistently higher than those of SUV39H1 (Fig. 1b). Moreover, according to the nonparametric test of the data, we found that the expression level of SUV39H2 was correlated with the CNA of the gene (Fig. 1c), which implies that the gain of copy number in tumor cells might be the underlying mechanism that causes SUV39H2 overexpression. Furthermore, we employed the TCGA data for survival analysis to reveal the potential prognostic value underlying the SUV39H2 overexpression in lung cancer tissue. The results indicated that patients with higher SUV39H2 expression tended to suffer from poorer survival (Fig. 1d). We then employed univariate analysis to examine the relationship between the indicated factors and OS (Table 1), which exhibited that the SUV39H2 expression level, TNM staging, T staging, and N staging were univariately correlated with OS. Furthermore, we performed a multivariate analysis, which suggested that a higher SUV39H2 expression level (*p* = 0.016, HR = 1.564) and later TNM staging (*p* < 0.001, HR = 1.527) were associated with poorer prognosis.

**Table 1:** Univariate and multivariate analysis of variables correlated to OS

	Univariate analysis			Multivariate analysis		
	*p* value	HR	95% CI	*p* value	HR	95% CI
SUV39H2 expression	0.004	1.632	1.165–1.846	0.016	1.564	1.088–2.248
Gender	0.758	1.047	0.783–1.400			
Age	0.208	1.208	0.900–1.621			
Smoking status	0.547	0.919	0.699–1.209			
TNM staging	< 0.001	1.621	1.424–1.846	< 0.001	1.527	1.327–1.758
T staging	< 0.001	1.562	1.300–1.878			
N staging	< 0.001	1.721	1.451–2.040			

### SUV39H2 overexpression in lung cancer tissues and lung cancer cell lines potentially influenced the expression of a variety of genes

**Fig. 2:**
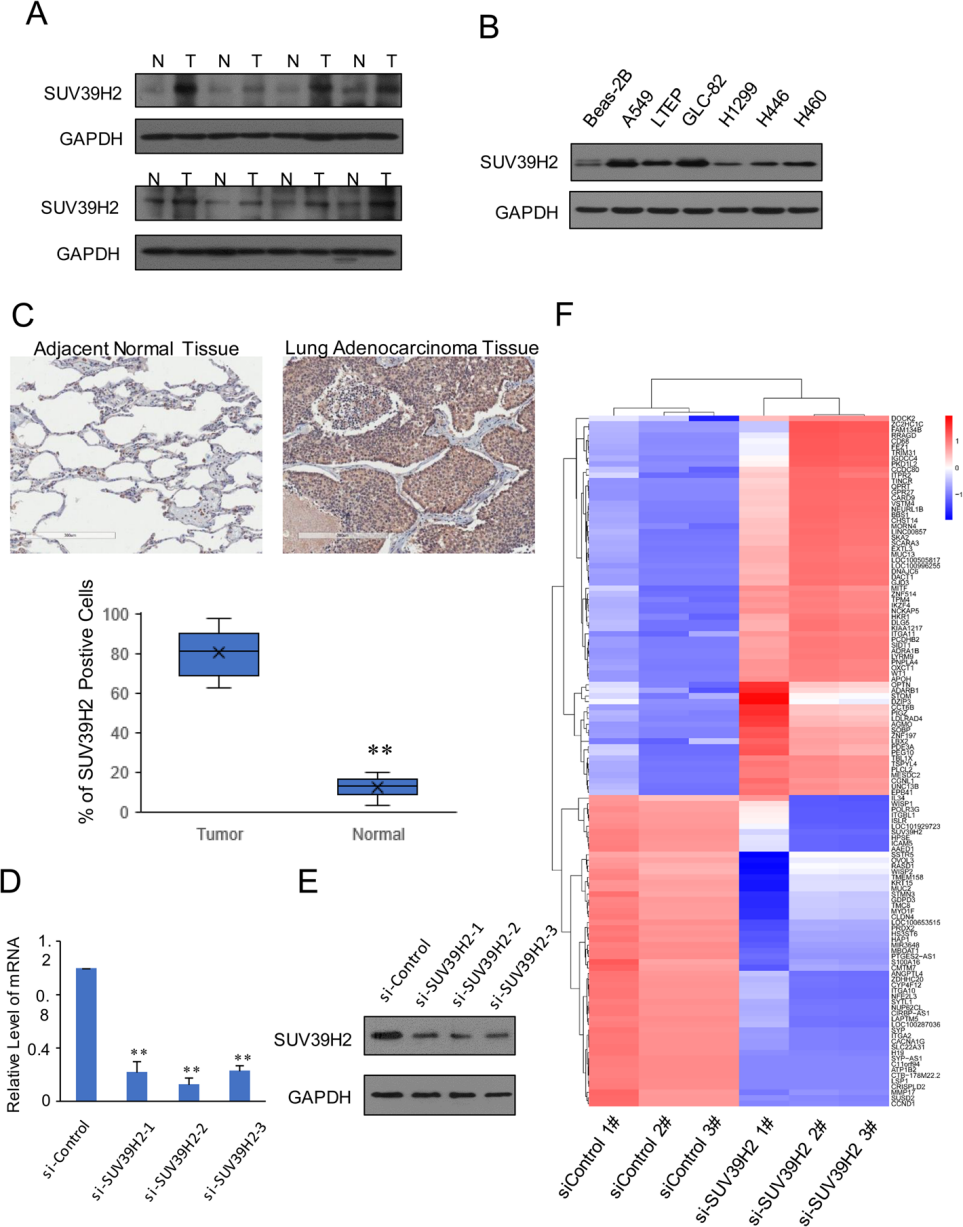
SUV39H2 overexpression in lung cancer tissues and lung cancer cell lines potentially influenced the expression of a variety of genes. **a** The globular proteins of eight surgically removed tumor tissues and the adjacent normal tissues were extracted, and the expression of SUV39H2 was assessed via Western blotting, with GAPDH serving as the internal control. **b** The whole-cell lysates of the indicated cell lines were used in the Western blot analysis to examine the expression of SUV39H2; GAPDH served as the control. **c** The representative image of SUV39H2 stain in indicated tissue sample of TMA and the percentage of SUV39H2-positive cells in each group are presented. **d**, **e** qRT-PCR and Western blotting were utilized to verify the knockdown of SUV39H2 at both the mRNA and protein levels. Each sample with the indicated primers was used for the PCR analyses in three replicates, and the experiment was performed in triplicate. **f** Heatmap of the representative genes identified in the RNA-seq. The samples were from three independent transfections with three different siRNA sequences

Since the overexpression of SUV39H2 was identified to be of importance in the online datasets, we then evaluated the expression of SUV39H2 in surgically removed NSCLC tissues via Western blotting, the results of which showed high consistency with those of the bioinformatics analyses (Fig. 2a). Given that SUV39H2 introduces H3K9me3 to the histone and mediates transcriptional repression [4], what the underlying mechanisms or the specific target genes of SUV39H2 could be is more intriguing. Hence, the expression spectrum analysis of SUV39H2 in lung cancer cell lines was performed, and SUV39H2 overexpression was seen in cells of A549, LTEP, and GLC-82, all of which are lung adenocarcinoma cell lines (Fig. 2b). Then, we performed immunohistochemistry in a tissue micro-array (TMA), which was composed of 32 lung adenocarcinoma and 12 normal adjacent tissue samples. Results demonstrated that SUV39H2 expression was significantly elevated in tumor tissue samples (Fig. 2c). Next, we utilized small interfering RNA to specifically knock down SUV39H2 in A549 cells, followed by the validation of knockdown efficiency through RT-qPCR and Western blotting (Fig. 2d, e). Then, the mRNA of the indicated A549 cells was extracted and subjected to next-generation sequencing (NGS); the representative upregulated and downregulated genes were plotted in the heatmap (Fig. 2f), from which we may conclude that the knockdown of SUV39H2 resulted in a significant transcriptome alteration.

### Identification of potential target genes of SUV39H2

**Fig. 3:**
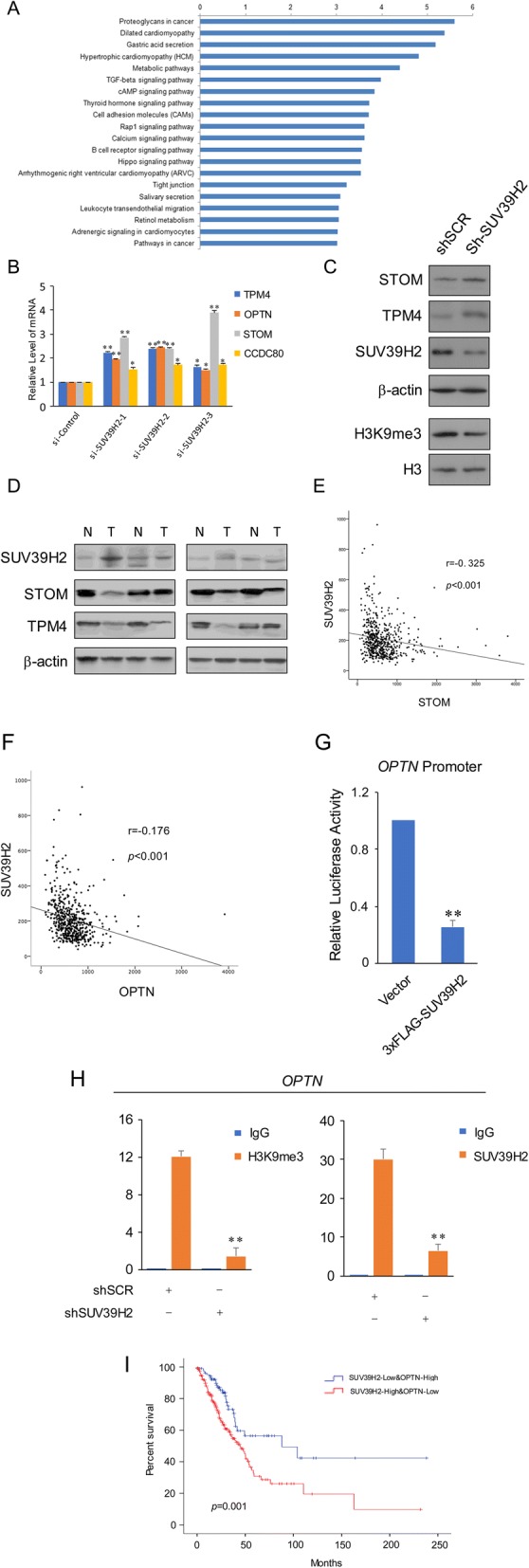
Identification of the potential target genes of SUV39H2. **a** The pathway enrichment assay was conducted according to the KEGG pathway database, and the top 20 pathways in the assay are enlisted in the figure. **b** mRNA expression of the indicated genes in control or SUV39H2 knockdown A549 cells. The error bars represent the mean ± SD of three independent experiments. *p < 0.05, **p < 0.01 (two-tailed unpaired *t* test). Each sample with the indicated primers was used for the PCR analyses in three replicates, and the experiment was performed in triplicate. **c** The protein level of STOM and TPM4 and H3K9me3 level in SUV39H2 knockdown A549 cells. **d** The protein level of STOM and TPM4 in surgically removed lung adenocarcinoma tissue samples and their paired normal adjacent tissue samples. **e**, **f** The correlation analysis of SUV39H2 with OPTN or STOM with regard to mRNA expression. **g** The OPTN promoter-mediated luciferase activity in indicated cells. Each group was composed of six samples and each experiment was repeated three times. **h** The ChIP-qPCR assay conducted with indicated antibodies in each group of cells. **p < 0.01. **i** Kaplan-Meier plots of overall survival associated with mRNA expression levels of SUV39H2 and OPTN in TCGA lung adenocarcinomas

A pathway analysis was performed against the NGS data. The results suggested that knockdown of SUV39H2 might have an impact on a series of pathways including metabolism in cancer, TGF-β signaling pathway, Hippo signaling pathway, and pathways in cancer (Fig. 3a). Among all the genes with altered expressions, we identified OPTN and STOM, which were also downregulated in the previous analysis and participated in tumorigenesis. Moreover, we found TPM4 [41, 42] and CCDC80 [43, 44], which were reported to exert a tumor repression activity, to be upregulated due to SUV39H2 knockdown. Then, we conducted RT-qPCR analysis to validate the potential regulation of the four genes by SUV39H2. Results showed that all these four genes were significantly upregulated in SUV39H2-knockdown A549 cells (Fig. 3b). Furthermore, we tested the protein change of STOM and TPM4 in SUV39H2 steadily knockdown A549 cells, and the results indicated the same as that from the RT-qPCR assay (Fig. 3c). Moreover, we collected four tissue samples of surgically removed lung adenocarcinoma and their paired normal adjacent tissue to evaluate the protein level of STOM and TPM4 (Fig. 3d). To further consolidate the potential relationship, we again utilized the TCGA dataset and analyzed the relationship between SUV39H2 and OPTN or STOM via the Spearman correlation analysis. The results show that the expression of SUV39H2 was significantly negatively correlated with OPTN (*r* = − 0.176, *p* < 0.001) and STOM (*r* = − 0.325, *p* < 0.001) (Fig. 3e, f). As stated previously, OPTN could suppress the growth and tumorigenicity of lung cancer cells [35]. As the negative correlation between OPTN and SUV39H2 was strongly indicated, we employed luciferase assay against the promoter of *OPTN*, which suggested that overexpression of SUV39H2 significantly repressed the luciferase activity via interacting with the promoter of *OPTN* (Fig. 3g). Then, we performed chromatin immunoprecipitation (ChIP) to further validate the transcriptional regulation of SUV39H2 against *OPTN*. The results demonstrated that loss of SUV39H2 not only diminished the enrichment of SUV39H2 but also the enrichment of H3K9me3 on the promoter of *OPTN* (Fig. 3h), so we primarily identified the transcriptional repression of OPTN mediated by SUV39H2. Finally, we grouped the patients according to the expressions of both SUV39H2 and OPTN as indicated and determined the overall survival (OS) in the two groups (Fig. 3i). The Kaplan-Meier plot demonstrated that patients with a high level of SUV39H2 expression and low level of OPTN expression tended to suffer from shorter OS than patients with a low level of SUV39H2 expression (*p* = 0.001).

### Potential function of SUV39H2 in lung adenocarcinoma cell lines

**Fig. 4:**
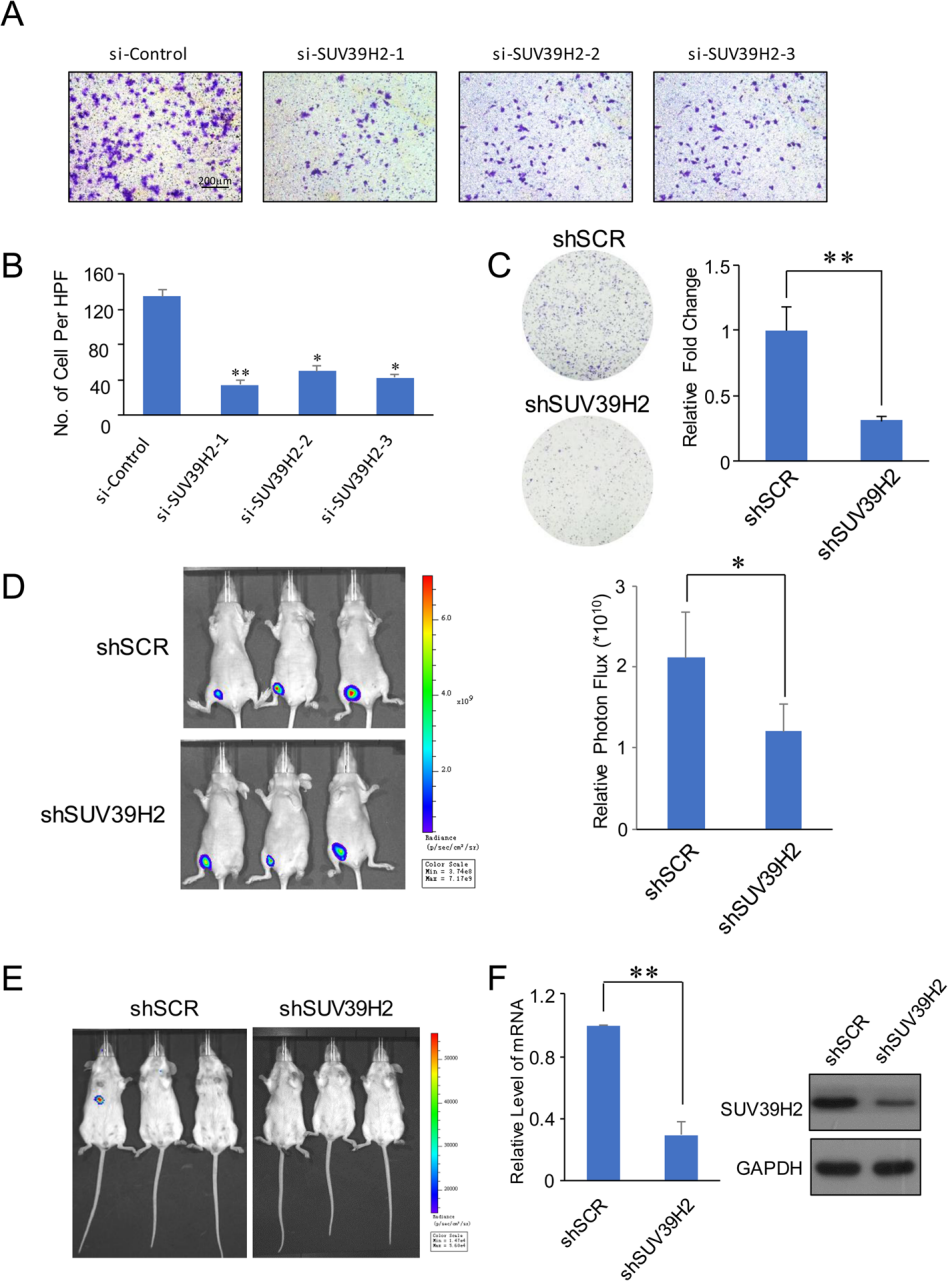
Potential function of SUV39H2 in lung adenocarcinoma cell lines. **a**, **b** A549 cells were transfected with siControl or siRNA targeting SUV39H2, before being seeded into the transwell chambers. The invaded cells were stained and counted, and the images represent one field under the microscope (× 10 magnification). The error bars represent the mean ± SD of three independent experiments. *p < 0.05, **p < 0.01 (two-tailed unpaired *t* test). For each complete experiment, three independent samples were subjected to analysis in the indicated group, and every experiment was conducted in triplicate. **c** A549 cells infected with the indicated lentiviruses were maintained in culture media for 5 days prior to being stained with crystal violet. Representative photos are shown on the left; they were statistically analyzed as shown on the right. For each complete experiment, three independent samples were analyzed in the indicated group, and every experiment was conducted in triplicate. **d** A549 cells infected with lentiviruses expressing shSCR or shSUV39H2 were inoculated subcutaneously in 6-week-old female nude mice (*n* = 6). Six tumors were quantified using bioluminescence imaging 1 week after the initial implantation. The error bars indicate the mean ± SD. *p < 0.05, **p < 0.01 (two-tailed *t* test). **e** A549 cells infected with lentiviruses expressing shSCR of shSUV39H2 were injected intravenously in 6-week-old female SCID mice (*n* = 3). The tumors were detected via bioluminescence imaging. **f** The knockdown efficiency was confirmed by RT-qPCR and Western blotting

As shown in the pathway analysis, the cell junction pathway was identified to be dysregulated, and we performed the invasion assay in A549 cells with or without SUV39H2 knockdown. Results showed that the invasion of the SUV39H2 knockdown cells was severely inhibited, suggesting a prominent role of SUV39H2 in promoting A549 cell invasion (Fig. 3a, b). Due to the potential regulation of SUV39H2 over OPTN and the tumor-repressive activity of OPTN, we next conducted clone formation assays in the A549 cells with SUV39H2 knockdown. As demonstrated in Fig. 4c, the clone formation activity of A549 cells with SUV39H2 knockdown was severely impaired. Next, we investigated the role of SUV39H2 in tumor progression in vivo. For this purpose, we performed a bioluminescence assay to measure tumor growth in situ in nude athymic BALB/c mice or in immunocompromised SCID mice. A549 cells stably expressing luciferase were infected with the indicated lentiviruses and seeded either subcutaneously or intravenously in nude athymic BALB/c mice or in immunocompromised SCID mice, respectively, and the bioluminescence of the neoplasia was measured via the IVIS imaging system (Xenogen). As shown in Fig. 4d, the knockdown of SUV39H2 severely impaired the tumor growth in situ. Additionally, in the intravenous injection group, liver metastasis was significantly abrogated in the SUV39H2 knockdown group (Fig. 4e). Consistently, both the in vitro and in vivo assays demonstrated that the loss of function of SUV39H2 resulted in suppressed tumor growth and invasion. The validation of SUV39H2 knockdown is shown in Fig. 4f.

## Discussion

**Fig. 5:**
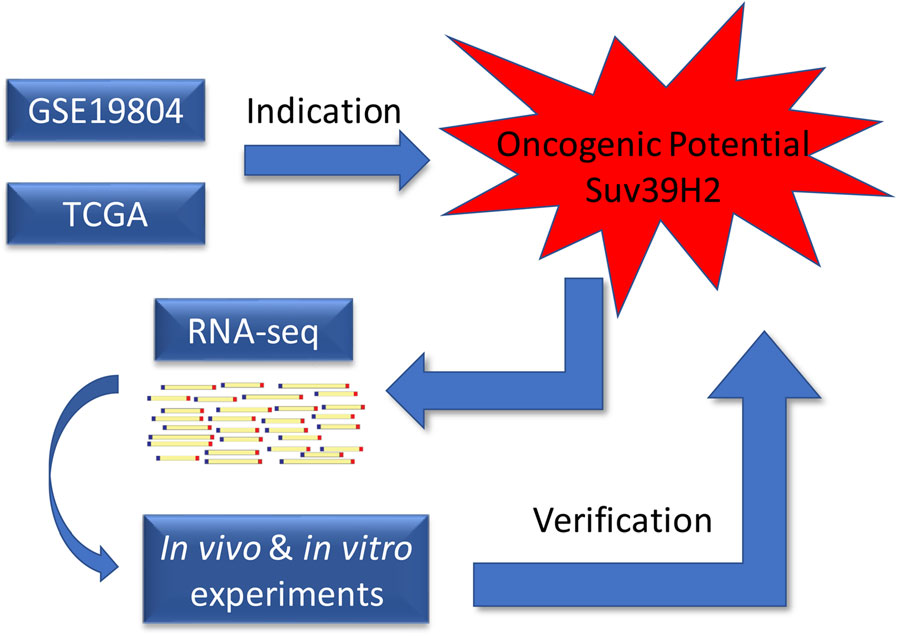
The work flow and experiment design of the research

In this research, we analyzed the chip assay of lung adenocarcinoma samples and identified SUV39H2 as a potential oncogene due to its overexpression in the tumor tissues. Because of its emerging role in cancers [18, 22, 45], the underlying mechanisms are becoming the focus of studies about SUV39H2. Since it plays a role in regulating the high-order chromatin dynamics [8], the transcriptional repression induced by SUV39H2 might be a major molecular basis for its overexpression. Therefore, we mainly focused on the downregulated genes in the RNA-seq results and identified OPTN, STOM, TPM4, and CCDC80 as potential target genes (Fig. 5). Moreover, we failed to identify SUV39H1 as a differentially expressed gene in the chip analysis, indicating that it might have a unique role in carcinogenesis. We also probed the molecular basis that might cause the specific overexpression of SUV39H2 in tumor cells. Compared to SUV39H1, the amplification percentage of SUV39H2 was much higher throughout the various stages of lung adenocarcinoma; guided by which, we found that the CNA and the expression of SUV39H2 shared a positive relation, leading to an explanation of its overexpression [46]. Furthermore, we also uncovered the overexpression of some classical oncogenes (FOXA1, PCNA, EZH2) that were reported to mediate carcinogenesis in lung adenocarcinoma along with SUV39H2.

We selected the most case-abundant dataset [25, 26], and the survival analysis strongly suggested the oncogenic role of SUV39H2. Furthermore, the statistical analysis again verified its correlation with the overall survival. The cell line assay of SUV39H2 expression showed that SUV39H2 was overexpressed to a greater degree in lung adenocarcinoma than in squamous cell carcinoma, which might also explain the fact that we did not find a notable benefit from the low expression of SUV39H2 in squamous cell carcinoma (TCGA, Provisional) (data not shown).

Moreover, after the primary analysis of the online datasets, we managed to further clarify the oncogenic potential of SUV39H2 via RNA-seq. Through pathway enrichment, we found that several important pathways including metabolism in cancer [47], TGF-β signaling [48], and cell junction [49], all of which are involved in tumorigenesis and progression, are druggable targets during cancer treatment. We also identified several potential target genes of SUV39H2 due to their elevated expressions in the context of SUV39H2 knockdown; however, the underlying transcriptional regulation is still unclear and worth probing.

In addition, further ChIP assays of H3K9me3 indicated that the knockdown of SUV39H2 resulted in the impairment of H3K9me3 enrichment on the promoter of the OPTN, implicating that SUV39H2 could still have an impact on the transcriptional regulation of OPTN. However, the detailed molecular mechanism is still unclear. Analogously, SUV39H2 was reported to be recruited on the promoter of SLIT1 and promote the proliferation and metastasis of colorectal cancer [50], suggesting that the epigenetic modulation of SUV39H2 was of importance. Moreover, histone demethylase LSD1 was found to be stabilized by SUV39H2 via methylation [45], which raised another hypothesis that SUV39H2 correlates with LSD1 during the transcription regulation.

Since the utilization of methyltransferase-targeted therapy is becoming increasingly important in cancer treatment [51, 52], this research illustrated the potential oncogenic activity of SUV39H2 in lung adenocarcinoma and the possible target genes, which provided a deeper insight into its role in carcinogenesis. The inhibitors targeting EZH2, which is co-overexpressed with SUV39H2 in lung cancer, are being thoroughly researched [53–55]. This research provides strong clues that SUV39H2 can be a potential drug target in lung cancer and a candidate indicator of cancer prognosis.

## Conclusions

In this study, we discovered that SUV39H2 is a potential oncogene in lung adenocarcinoma, whose expression was elevated in tumor tissues. SUV39H2 could potentiate the tumorigenesis and invasion of lung adenocarcinoma cells, probably by repressing OPTN and STOM.

## Additional files

Additional file 1: **Table S1.** Analysis of the chip assay GSE19840. (XLSX 624 kb)

Additional file 2: **Table S2.** Primers used in the qRT-PCR assay. (DOCX 13 kb)

Additional file 3: **Table S3.** siRNA and shRNA. (DOCX 14 kb)

## Abbreviations

EHMT1: Euchromatic histone lysine methyltransferase 1
EHMT2: Euchromatic histone lysine methyltransferase 2
EZH2: Enhancer of zeste 2 polycomb repressive complex 2 subunit
FOXA1: Forkhead box A1
KMT: Histone lysine methyltransferase
NSCLC: Non-small cell lung carcinoma
OPTN: Optineurin
PCNA: Proliferating cell nuclear antigen
SETDB1: SET domain bifurcated 1
SETDB2: SET domain bifurcated 2
STOM: Stomatin
SUV39H2: Suppressor of variegation 3-9 homolog 2
TCGA: The Cancer Genome Atlas
TPM4: Tropomyosin 4
