# Supplementary material for: Identification of SUV39H2 as a potential oncogene in lung adenocarcinoma
Source: Clin Epigenetics. 2018 Oct 22;10:129. doi: 10.1186/s13148-018-0562-4 (PMC6198372; doi:10.1186/s13148-018-0562-4)
Supplement: Supplementary file 2 — Table S2. Primers used in the qRT-PCR assay. (DOCX 13 kb) [file 13148_2018_562_MOESM2_ESM.docx]

| Gene | Strand | Sequence (5’-3’) |
| --- | --- | --- |
| SUV39H2 | F | ATTGATAACCTCGATACTCGTCTT |
| SUV39H2 | R | TCTCCAGAACCTTTCATTTGATAA |
| OPTN | F | GGAGAAGCAGAAGGGTCAGT |
| OPTN | R | TTTAGACAATGCCGTGCCAG |
| STOM | F | ACACAACATGCAGTCTACTCT |
| STOM | R | GTTTCACATCCTTAATTTCCACAC |
| TPM4 | F | AAACTGAAAGAGGCTGAGACC |
| TPM4 | R | GCAAGTTTCTCTTCCAGGTCAT |
| CCDC80 | F | GAGAGTCAAGCAATACTATGAGG |
| CCDC80 | R | AAACAATGCCCTCCTTCTTCT |
| β-actin | F | TGGCACCCAGCACAATGAA |
| β-actin | R | CTAAGTCATAGTCCGCCTAGAAGCA |
