# Supplementary material for: Identification of SUV39H2 as a potential oncogene in lung adenocarcinoma
Source: Clin Epigenetics. 2018 Oct 22;10:129. doi: 10.1186/s13148-018-0562-4 (PMC6198372; doi:10.1186/s13148-018-0562-4)
Supplement: Supplementary file 3 — Table S3. siRNA and shRNA. (DOCX 14 kb) [file 13148_2018_562_MOESM3_ESM.docx]

|  | Sequence (5’-3’) |
| --- | --- |
| shSCR | TTCTCCGAACGTGTCACGT |
| shSUV39H2 | GGCTAAACAAAGGATAGCTCT |
| si-Control | UUCUCCGAACGUGUCACGUTT |
| si-SUV39H2-1 | GCAUCUUUCGAACUAGCAATT |
| si-SUV39H2-2 | GACAACAAGGGAAUCACGUTT |
| si-SUV39H2-3 | CAUUAACGAAUACAAACCATT |
